# Metastatic Pancreatic Cancer: Where Are We?

**DOI:** 10.3389/or.2023.11364

**Published:** 2024-01-18

**Authors:** Abraham Hernández-Blanquisett, Valeria Quintero-Carreño, María Cristina Martínez-Ávila, María Porto, María Carolina Manzur-Barbur, Emiro Buendía

**Affiliations:** ^1^ Cancer Institute, Hospital Serena del Mar, Cartagena, Colombia; ^2^ Clinical Oncology, Hospital Serena del Mar, Cartagena, Colombia; ^3^ Pain and Palliative Care Department, Hospital Serena del Mar, Cartagena, Colombia; ^4^ Internal Medicine Department, Hospital Serena del Mar, Cartagena, Colombia

**Keywords:** chemotherapy, pancreatic cancer, metastatic pancreatic cancer, molecular targeted therapy, supportive care

## Abstract

Pancreatic cancer is one of the most lethal neoplasms worldwide; it is aggressive in nature and has a poor prognosis. The overall survival rate for pancreatic cancer is low. Most patients present non-specific symptoms in the advanced stages, which generally leads to late diagnosis, at which point there is no option for curative surgery. The treatment of metastatic pancreatic cancer includes systemic therapy, in some cases radiotherapy, and more recently, molecular targeted therapies, which can positively impact cancer control and improve quality of life. This review provides an overview of the molecular landscape of pancreatic cancer based on the most recent literature, as well as current treatment options for patients with metastatic pancreatic cancer.

## Introduction

Pancreatic cancer (PC) is an aggressive disease, classified as one of the highest lethal malignancies [1]. PC is the 10th most common cancer but currently ranks as the third leading cause of cancer-related death in the United States and represents the fourth leading cause of cancer death worldwide [1–3]. Risk factors for developing CP include somatic genetic aberrations that occur throughout one’s lifetime and can be precipitated by tissue injury and influenced by germline risk variants, immune response, and other factors [2]. The lifetime risk for PC is estimated at 1.7% for the “average” individual; this risk may be increased by factors such as cigarette smoking, diabetes, alcohol, obesity, pancreatitis, intraductal papillary mucinous neoplasms and mucinous cystic neoplasms of the pancreas, Lynch syndrome, Peutz-Jeghers syndrome, familial pancreatic cancer, familial atypical multiple mole melanoma syndrome, and inherited susceptibility [2, 4]. Although we are in a new era of oncology, where molecular target-directed therapy has dramatically changed the prognosis of some types of cancer, such as lung and ovarian cancer, CP continues to be a disease with a poor prognosis and few treatment options. The lack of cardinal symptoms leads to late diagnosis, as most patients present with unresectable disease due to locally advanced involvement (30%) or metastatic disease (50%) at the time of diagnosis [5]. For metastatic pancreatic cancer (MPC), treatment with systemic chemotherapy plays an important role, as it remains the best management option to increase survival, alleviate symptoms, and ensure better quality of life (QoL) [6, 7]. The management of MPC represents one of the main challenges for clinical oncologists and the need for new strategies is more evident in the context of molecular target-driven medicine. This article provides an overview of the molecular landscape of PC and current treatment options for MPC patients based on the most recent literature.

## Molecular Landscape

PC presents a large number of mutations and somatic copy number alterations (SCNAs) that can alter the function of oncogenes and tumor suppressor genes, including KRAS, TP53, SMAD4, and CDKN2A [8]. KRAS mutation is an early oncogenic event in PC because it is detectable in up to 38% of premalignant pancreatic lesions, indicating that KRAS mutation is likely an early and initiating event in PC [9]. Approximately 93% of PCs could have KRAS somatic mutations, and multiple KRAS oncogenic alleles have been identified, the most frequent being KRAS G12D, G12V, and G12R, while the KRAS G12C mutation is much less frequent and is present in 1.3% of PCs [10]. Approximately 10% of PC harbor germline or somatic mutations in one of the ATM, BRCA 1, BRCA1, and PALB 2 DNA repair genes, and there is a very low prevalence of alterations in BRAF, PIK3CA, RNF43, STK11, JAK1, and ERBB2 genes [11].

Most cases of PC are sporadic, but it can be associated with Lynch syndrome (LS). Patients with LS have an 8.6-fold higher risk than the general population of developing PC and a cumulative risk of 3.7% at 70 years [12]. In LS, one allele of one of the genes that govern mismatch repair (MLH1, MSH2, MSH6, and PMS2) is mutated in the germline and a second mutation occurs spontaneously, whereas in sporadic cases, one allele is spontaneously mutated and the second is epigenetically silenced [13].

The American Society of Clinical Oncology (ASCO) guidelines, in their latest 2020 version, strongly recommend performing both germline and tumor (somatic) early testing to detect actionable genomic alterations in patients who are likely potential candidates for additional treatment after first-line therapy. These tests include microsatellite instability/mismatch repair deficiency testing, BRCA mutations (excluding variants of unknown significance), and NTRK gene fusions [14].

## Chemotherapy for Metastatic Pancreatic Cancer

### First-Line Treatment

Based on evidence from randomized clinical trials, there is now a clear role for chemotherapy in metastatic and unresectable disease, showing increased survival benefits, but it also has the negative effect of increased toxicity (Table 1).

**TABLE 1:** Chemotherapy for metastatic pancreatic cancer first line treatment.

Study	Population	Experimental arm	Control arm	Outcomes	Reference
Single-agent gemcitabine	126 patients with diagnosis of PC that was locally advanced or metastatic. Karnofsky score at least 50%.	Gemcitabine 1,000 mg/m^2^ weekly for up to 7 weeks followed by a week of rest, then weekly for 3 consecutive weeks out of Q4W	5-fluorouracil (5-FU) at 600 mg/m^2^ IV weekly, with a cycle defined as one 4 week period	Clinical Benefit response 23.8% vs. 4.8%	[11]
				OS 5.6 months vs. 4.4 months	
				OS12: 18% vs. 2%	
				TTP 9 weeks vs. 4 weeks	
				ORR 5.4% vs. 0%	
NCT00026338	569 patients with an ECOG status of 0, 1 or 2 diagnosed with advanced stage PC or metastatic disease that is considered unresectable	Gemcitabine 1,000 mg/m^2^ weekly for up to 7 weeks followed by a week of rest, then weekly for 3 consecutive weeks out of Q4W plus Erlotinib 150 mg OD	Gemcitabine 1,000 mg/m^2^ weekly for up to 7 weeks followed by a week of rest, then once weekly for 3 consecutive weeks out of Q4W plus placebo	OS 6.24 months vs. 5.91 months	[12]
				OS12 23% VS 17%	
				PFS 3.75 months vs. 3.55 months	
				DOR 163 days both arms	
NCT00112658	342 patients younger than 76 years with an ECOG PS score of 0 or 1 diagnosed with metastatic and locally advanced PC	FOLFIRINOX: oxaliplatin, 85 mg/m^2^; irinotecan, 180 mg/m^2^; leucovorin, 400 mg/m^2^; and 5-FU 400 mg/m^2^ bolus followed by 2,400 mg/m^2^ given as a 46 h continuous infusion, Q2W	Gemcitabine 1,000 mg/m^2^ weekly for up to 7 weeks followed by a week of rest, then weekly for 3 consecutive weeks out of Q4W	OS 11.1 months vs. 6.8 months	[14]
				OS12 48.4% vs. 20.6%	
				PFS 6.4 months vs. 3.3 months	
				ORR 31.6% vs. 9.4%	
				DOR 5.9 months vs. 3.9 months	
GEST	832 chemotherapy-naive patients with locally advanced or MPC and with an ECOG PS score of 0–1	S-1: 80, 100, or 120 mg/d according to body-surface area on days 1 through 28 of a 42 day cycle GS: gemcitabine 1,000 mg/m^2^ on days 1 and 8 plus S-1 60, 80, or 100 mg/d according to body-surface area on days 1 through 14 of a 21 day cycle	Gemcitabine 1,000 mg/m^2^ on days 1, 8, and 15 of a 28 day cycle	OS 9.7 months vs. 10.1 months vs. 8.8 months	[15]
				OS12 38.5% vs. 40.7% vs. 35.4%	
				PFS 3.8 months vs. 5.7 months vs. 4.1 months	
				ORR 21% vs. 29.3% vs. 13.3%	
				DOR 2.6 months vs. 4.3 months vs. 2.6 months	
NCT00032175	533 patients with an ECOG PS of 0, 1, or 2 and diagnosis of locally advanced or MPC	Gemcitabine 1,000 mg/m^2^ weekly for up to 7 weeks followed by a week of rest, then weekly for 3 consecutive weeks out of Q4W plus capecitabine 1,830 mg/m^2^ bid for 3 weeks followed by 1 week’s rest	Gemcitabine 1,000 mg/m^2^ weekly for up to 7 weeks followed by a week of rest, then weekly for 3 consecutive weeks out of Q4W	OS 7.1 months vs. 6.2 months	[16]
				OS12 24.3% vs. 22%	
				PFS 5.3 months vs. 3.8 months	
				ORR: 19.1% vs. 12.4%	
MPACT	861 patients younger than 79 years with Karnofsky PS score of 70% or more and MPC	nab-paclitaxel 125 mg/m^2^ IV in combination with gemcitabine 1,000 mg/m^2^ weekly for 3 consecutive weeks out of Q4W	Gemcitabine 1,000 mg/m^2^ weekly for up to 7 weeks followed by a week of rest, then weekly for 3 consecutive weeks out of Q4W	OS 8.5 months vs. 6.7 months	[17]
				OS12 35% vs. 22%	
				PFS 5.5 months vs. 3.7 months	
				ORR 23% vs. 7%	
POLO	154 (92 to receive olaparib and 62 to receive placebo)	First line cisplatin, carboplatin or oxaliplatin plus olaparib tablets (300 mg twice daily) 4–8 weeks after the last dose of first-line chemotherapy	Matching placebo	PFS 7.4 months vs. 3.8 months	[18]
				OS median, 18.9 months vs. 18.1 months	
RESOLVE	213 patients (211 TO ibrutinib plus nab-paclitaxel/gemcitabine and 213 to placebo plus nabpaclitaxel/gemcitabine	Ibrutinib 560 mg once daily in combination with IV nab-paclitaxel (125 mg/m^2^) and IV gemcitabine (1,000 mg/m^2^) on day 1, 8, and 15 of each 28 day cycle	Placebo 560 mg once daily in combination with IV nab-paclitaxel (125 mg/m^2^) and IV gemcitabine (1,000 mg/m^2^) on day 1, 8, and 15 of each 28 day cycle	OS median of 9.7 versus 10.8 months	[19]
				PFS median 5.3 versus 6.0 months	
PRINCE	99 (32 to receive nivo/chemo, 31 to sotiga/chemo; 31 sotiga/nivo/chemo)	1,000 mg/m^2^ intravenous gemcitabine and 125 mg/m^2^ intravenous nab-paclitaxel, receiving as an experimental intervention 0·1 mg/kg intravenous Sotigalimab in two cohorts (B1 and C1) and 0·3 mg/kg in cohorts (B2 and C2), being cohorts C1 and C2 also treated with 240 mg intravenous nivolumab	1,000 mg/m^2^ intravenous gemcitabine and 125 mg/m^2^ intravenous nab-paclitaxel plus 240 mg intravenous nivolumab	OS for nivo/chemo 57.7%, compared to historical 1 year OS of 35%	[20, 21]
				OS for sotiga/chemo 48.1%	
				OS for sotiga/nivo/chemo 41.3%	

Abbreviations: OS, overall survival; OS12, 12-month overall survival; ORR, objective response rate; PFS, progression-free survival; PS, performance status; PC, pancreatic cancer; MPC, metastatic pancreatic cancer; DOR, duration of response; TTP, time to progression disease; ECOG, Eastern Cooperative Oncology Group; Q2W, every 2 weeks; Q4W, every 4 weeks; O, once daily; BID, orally twice a day.

#### Single-Agent Chemotherapy: Gemcitabine

Gemcitabine has emerged as the first alternative to 5-Fluorouracil (5-FU) [22]. The data came from a phase 2 trial that included 126 patients with PC that was locally advanced, unresectable, or metastatic. Intravenous gemcitabine 1,000 mg/m^2^ once a week for up to 7 weeks, followed by a week of rest, then once weekly for 3 consecutive weeks of every 4 weeks was compared with bolus 5-FU 600 mg/m^2^ IV once a week [23]. This treatment showed benefits in terms of clinical benefit response. In total, 23.8% of gemcitabine-treated patients experienced a clinically beneficial response compared to 4.8% of 5-FU-treated patients (*p* = 0.0022). The ORR was 5.4% in the gemcitabine arm and 0% in the 5-FU arm. There was a statistically significant improvement in the survival of patients who received gemcitabine (median overall survival -OS- 5.6 months, 18% 1 year survival versus median OS 4.4 months, 2% 1 year survival with 5-FU, *p* = 0.0025) [23].

#### Multiagent Chemotherapy

##### CAN-NCIC-PA3/NCT00026338: Erlotinib Plus Gemcitabine vs. Gemcitabine Alone

The addition of erlotinib was associated with a 10 day survival benefit; due to its additional toxicity, this regimen has not been widely used. The phase 3 CAN-NCIC-PA3/NCT00026338 trial was designed to evaluate the effects of adding erlotinib to gemcitabine in patients with unresectable, locally advanced, or metastatic PC [24]. In this trial, 569 patients with ECOG PS 0, 1, or 2 were randomly assigned to receive intravenous gemcitabine plus Erlotinib (150 mg/d) or matching placebo. Outcome was better in the erlotinib plus gemcitabine arm (23% 1 year survival, median OS 6.24 months for the erlotinib plus gemcitabine arm vs. 17% 1 year survival and median OS 5.91 months in the placebo plus gemcitabine group; *p* = 0.023), with an estimated HR of 0.82 (95% CI, 0.69 to 0.99; *p* = 0.038) [24]. However, toxicity was higher in the erlotinib group. The most common adverse events reported for the erlotinib plus gemcitabine combination were diarrhea (56%), fatigue (89%), rash (72%), and stomatitis (23%).

##### PRODIGE 4/ACCORD 11: FOLFIRINOX vs. Gemcitabine Alone

FOLFIRINOX combination regimen was assessed in the PRODIGE 4/ACCORD 11 (NCT00112658) trial, FOLFIRINOX (oxaliplatin, 85 mg/m^2^; irinotecan 180 mg/m^2^, leucovorin (LV) 400 mg/m^2^, and 5-FU 400 mg/m^2^ given as a bolus followed by 2,400 mg/m^2^ given as a 46-h continuous infusion every 2 weeks) was compared to gemcitabine at a 1,000 mg/m^2^ dose weekly for 7 of 8 weeks and then weekly for 3 of 4 weeks [25]. The primary outcome was OS. Median OS was 11.1 months (95% CI, 9.0–13.1) in the FOLFIRINOX group compared with 6.8 months (95% CI, 5.5–7.6) in the gemcitabine group (HR death, 0.57; 95% CI, 0.45–0.73). (*p* < 0.001) [25]. Median PFS was 6.4 months (95% CI, 5.5–7.2) in the FOLFIRINOX group and 3.3 months (95% CI, 2.2–3.6) in the gemcitabine group (HR for disease progression, 0.47; 95% CI, 0.37 to 0.59; *p* < 0.001). ORR was 31.6% (95% CI, 24.7–39.1) in the FOLFIRINOX group and 9.4% (95% CI, 5.4–14.7) in the gemcitabine group (*p* < 0.001) [25]. Nevertheless, FOLFIRINOX was associated with a higher incidence of grade 3 or 4 neutropenia (45.7%), febrile neutropenia (5.4%), thrombocytopenia (9.1%), diarrhea (12.8%), and sensory neuropathy (9.0%), as well as grade 2 alopecia [25]. Therefore, in daily practice, this treatment is preferred only in young patients who have a good performance status. Recently, some authors suggested that female gender could positively predict response to FOLFIRINOX in patients with advanced PC [15]. However, this topic deserves further evaluation [16].

##### GEST: Gemcitabine Plus S-1, S-1 Alone, or Gemcitabine Alone

Starting in 2007, a three-arm randomized phase 3 trial was conducted to assess whether S-1 alone is non-inferior to gemcitabine and whether gemcitabine plus S-1 (G-S-1) is superior to gemcitabine alone for locally advanced and metastatic PC with respect to OS [17]. In this trial, NCT00498225, 832 chemotherapy-naïve patients with locally advanced or metastatic PC and an ECOG PS of 0–1 were assigned to receive gemcitabine alone, S-1 monotherapy, or Gemcitabine-S-1 (gemcitabine 1,000 mg/m^2^ on days 1 and 8 plus S-1 60, 80, or 100 mg/d based on body surface area on days 1 through 14 of a 21 day cycle) [17]. Non-inferiority of S-1 vs. gemcitabine was demonstrated (HR, 0.96; 97.5% CI, 0.78 to 1.18; *p* < 0.001 for non-inferiority), while superiority of G-S-1 could not be demonstrated (HR, 0.88; 97.5% CI, 0.71 to 1.08; *p* = 0.15) [17]. Another subsequent study reported the long-term results of the GEST study and reconfirmed the non-inferiority of S-1 versus gemcitabine, demonstrating that S-1 can be considered as one of the treatment options for advanced PC [15, 18]; however, this therapeutic option is not available in most Western countries.

##### MPACT: Gemcitabine and Nab-Paclitaxel vs. Gemcitabine

The MPACT study (NCT00844649), a multicenter, phase 3 trial, was conducted 10 years ago, in 2013, to evaluate the efficacy and safety of the combination gemcitabine/nab-paclitaxel (Abraxane) vs. gemcitabine monotherapy in patients younger than 79 years with good PS (Karnofsky score of 70 or more) and metastatic PC [19]. A total of 861 patients were randomly assigned to receive nab-paclitaxel (125 mg/m^2^) followed by gemcitabine (1,000 mg/m^2^) on days 1, 8, and 15 every 4 weeks or gemcitabine monotherapy [19]. The median OS was 8.5 months in the nab-paclitaxel/gemcitabine group compared with 6.7 months in the gemcitabine group (HR death, 0.72; 95% CI, 0.62 to 0.83; *p* < 0.001). With respect to the secondary endpoints (PFS and ORR), there were significant improvements with nab-paclitaxel/gemcitabine. Median PFS was 5.5 months in the nab-paclitaxel/gemcitabine group and 3.7 months in the gemcitabine group (HR disease progression, 0.69; 95% CI, 0.58 to 0.82, *p* < 0.001). ORR was significantly higher with nab-paclitaxel/gemcitabine (23%; 95% CI, 19–27) than with gemcitabine (7%; 95% CI, 5–10) in the two groups (*p* < 0.001) [19]. The most common adverse events of grade 3 or higher were related to myelosuppression, neutropenia (38% in the nab-paclitaxel–gemcitabine group vs. 27% in the gemcitabine group), fatigue (17% vs. 7%), and peripheral neuropathy (17% vs. 1%); however, these side effects appear to be reversible [19]. These results established this combination as the new standard treatment for metastatic pancreatic cancer, especially since it is generally well tolerated by patients with ECOG 0 to 2. Due to this, even today, Gemcitabine associated with nab-paclitaxel continues to be the preferred regimen today, in addition to having a lower cost than new treatment options, facilitating greater access among low- and middle-income countries.

##### NCT00032175: Gemcitabine vs. Gemcitabine Plus Capecitabine

Taking into account the results of previous trials, a combination of gemcitabine and Capecitabine was evaluated in the GEM-CAP study, a multicenter, open-label, phase 3 trial [20]. In this trial, 533 patients with an ECOG PS of 0, 1, or 2 received gemcitabine by injection at 1,000 mg/m^2^ weekly for 7 weeks, followed by 1 week off, then weekly for 3 weeks every 4 weeks. Patients allocated to the GEM-CAP arm received gemcitabine intravenously at 1,000 mg/m^2^ weekly for 3 weeks every 4 weeks. Capecitabine was administered orally (830 mg/m^2^ twice daily) for 3 weeks followed by 1 week off [20]. GEM-CAP was associated with a significantly improved PFS over gemcitabine (HR, 0.78; 95% CI, 0.66 to 0.93; *p* = 0.004). The median PFS for GEM-CAP was 5.3 months (95% CI, 4.5–5.7), and for the gemcitabine group, it was 3.8 months (95% CI, 2.9–4.8). The median OS for GEM-CAP was 7.1 months, and for gemcitabine, it was 6.2 months (1-year survival 24.3% vs. 22%) [20]. However, this benefit is not clinically relevant.

##### POLO: First-Line Cisplatin, Carboplatin, or Oxaliplatin Plus Olaparib Maintenance vs. Placebo Maintenance

The POLO study (NCT02184195) compared the efficacy of Olaparib as maintenance therapy for advanced PC. Patients who harbored a BRCA1 or BRCA2 germline mutation and had a response or stable disease after first-line platinum-based chemotherapy were included [21]. Patients were randomly assigned, at a ratio of 3:2, to receive maintenance Olaparib tablets (300 mg twice daily) or a matching placebo, beginning 4–8 weeks after the last dose of first-line chemotherapy and continuing until the occurrence of objective radiologic disease progression. The results showed a clinical benefit compared to placebo (PFS: 7.4 months vs. 3.8 months; hazard ratio for disease progression or death, 0.53; 95% CI, 0.35 to 0.82; *p* = 0.004) [21]. An interim analysis of OS, at a data maturity of 46%, failed to demonstrate the differences between treatment arms (18.9 months vs. 18.1 months *p* = 0.68). Conveniently, there were no differences in health-related quality of life, and toxicity was acceptable for the Olaparib group. The incidence of grade 3 or higher adverse events was 40% in the treatment arm and 23% in the placebo group; 5% and 2% of the patients, respectively, discontinued the trial intervention for toxicity [21].

##### PRINCE: Sotigalimab and/or Nivolumab Plus Gemcitabine and Nab-Paclitaxel

Initially, the PRINCE study (NCT03214250) showed the safety of gemcitabine and nab-paclitaxel (chemo) plus Sotigalimab, a CD40 agonistic antibody (Sotigalimab/chemo), and/or nivolumab, an anti-PD-1 antibody (Sotigalimab/nivolumab/chemotherapy), using an open-label, multicenter, four-cohort, phase 1b study conducted at seven academic hospitals in the USA. his study included patients treated with 1,000 mg/m^2^ intravenous gemcitabine and 125 mg/m^2^ intravenous nab-paclitaxel, receiving as an experimental intervention 0·1 mg/kg intravenous APX005M in two cohorts (B1 and C1) and 0·3 mg/kg in two cohorts (B2 and C2), cohorts C1 and C2 also being treated with 240 mg intravenous nivolumab [26]. Later, a phase 2 study demonstrated better survival with nivolumab plus chemotherapy (1-y OS 57.7% vs. 35% historical 1-y OS *p* = 0.006) but failed to show differences with the Sotigalimab/Nivolumab/chemotherapy triplet (1-y OS 41.3%, *p* = 0.223) or Sotigalimab/chemotherapy (1-y OS 48.1%, *p* = 0.062). The authors reported some emerging immune signatures associated with survival for nivolumab/chemo and Sotigalimab/chemo. However, no biomarkers associated with a significant benefit for the triplet treatment were identified.

##### NAPOLI-3: NALIRIFOX

Nab-paclitaxel and gemcitabine is the standard first-line treatment for advanced PC in most countries. The NAPOLI 3 open-label, phase 3 trial compared this treatment against NALIRIFOX in patients with a good performance status; 383 subjects received NALIRIFOX, and 387 were included in the control arm [19]. Treatment arms consisted of NALIRIFOX (liposomal irinotecan 50 mg/m^2^, oxaliplatin 60 mg/m^2^, leucovorin 400 mg/m^2^, and fluorouracil 2,400 mg/m^2^, administered sequentially as a continuous intravenous infusion over 46 h) on days 1 and 15 of a 28-day cycle or nab-paclitaxel 125 mg/m^2^ and gemcitabine 1,000 mg/m^2^, administered intravenously on days 1, 8, and 15 of a 28 day cycle [19]. The trial found an OS benefit for NALIRINOX over nab-paclitaxel–gemcitabine (11.1 months versus 9.2 months HR: 0.83; *p* = 0.036). OS at 18 months was 26.2% and 19.3%, respectively. Median PFS was 7.4 months for NALIRIFOX and 5.6 for nab-paclitaxel and gemcitabine (HR 0.69; *p* < 0.0001) [19]. Despite being a statistically positive study, the clinical impact of this therapy is debatable, and the cost and tolerance seem to be a disadvantage compared to the current standard of treatment with gemcitabine and nab-paclitaxel. Another aspect to take into account is that we do not have data that compare the effectiveness of the FOLFIRINOX Scheme with NALIRINOX.

### Second-Line Treatment

Regarding second-line treatment, there are several treatment options for metastatic or locally advanced PC after gemcitabine failure. However, limited data exist and few phase 3 studies support any regimen (Table 2).

**TABLE 2:** Chemotherapy for metastatic pancreatic cancer second line treatment.

Study	Population	Experimental arm	Control arm	Outcomes	Reference
CONKO-003 part 1 NCT00786058	46 patients with advanced PC in progression confirmed by CT or MRI with Karnofsky performance status (KPS) >60%	OFF: oxaliplatin (85 mg/m^2^) on days 8 and 22, AF (200 mg/m^2^) followed by 5-FU infusion (2,000 mg/m^2^) administered on days 1, 8, 15 and 22. After a rest of 3 weeks the next cycle was started on d43	BSC	OS: 4.82 months vs. 2.30 months	[26]
CONKO-003 part 2	160 patients with diagnostic PC were stratified according to presence of metastases, duration of first-line gemcitabine therapy with Karnofsky performance status (KPS) of at least 70%	FF: folinic acid 200 mg/m2 IV infusion of fluorouracil 2,000 mg/m^2^ over 24 h on days 1, 8, 15, and 22	FF with OFF	OS: OFF 5.9 months vs. FF 3.3 months	[27]
		OFF: FF and oxaliplatin 85 mg/m2 IV administered before FF on days 8 and 22. After a 3 weeks rest periodfrom day 23–42, the second coursewas initiated on day 43		TTP: OFF 2.9 months vs. FF 2.0 months	
PANCREOX NCT01121848	108 patients with confirmed advanced PC who were previously treated with gemcitabine therapy and with an ECOG PS of 0–2 were eligible	FU/LV consisted of a dose of LV 400 mg/m^2^ administered as a 2-h IV infusion on day 1 and FU administered as a bolus IV dose of 400 mg/m^2^ on day 1 followed by a 2,400 mg/m^2^ continuous infusion for 46 h, administered every 14 days	mFOLFOX6 consisted of the same plus an oxaliplatin dose of 85 mg/m^2^ given as a 2-h IV infusion on day 1, administered every 14 days	No benefit was observed with the addition of oxaliplatin.	[28]
				PFS: 3.1 months vs. 2.9 months	
				OS: 6.1 months vs. 9.9 months	
NAPOLI-1 NCT01494506	417 patients with diagnostic PAM who have already been treated with gencitabine with Karnosfsky PS >70	First group received IN + FF (117 patients) IR in continuous infusion 80 mg/m^2^, 400 mg/m^2^ of FA and 2,400 mg/m^2^ of 5FU for 46 h every 2 weeks	The other group 149 patients) was treated with FA and 5FU received 200 mg/m^2^ of FA followed by 5FU for 24 h each week for the first 4 weeks of each 6 cycle. Patients on monotherapy treatment (151 patients) received 120 mg/m^2^ every 3 weeks IN	OS: 6.1 months vs. 4.2 months	
				OS: IN alone 4.9 months vs. 4.2 months	
KEYNOTE 158	It was 233 previously treated MSI-H/dMMR advanced cancer patients (27 different tumor types). Patients with previous exposure to immunotherapy were excluded	Pembrolizumab 200 mg administered intravenously every 3 weeks for 35 cycles or until disease progression or unacceptable toxicity	BSC	ORR: 34.3%%	[10]
				DOR: 13.4 months	
				PFS: 4.1 months	
				OS: 23.5 moths	
Sotorasib in KRAS p.G12C–Mutated	38 patients with KRAS p.G12C mutated advanced PC who had received at least one previous systemic therapy	Sotorasib a dose of 960 mg orally once daily	BSC	PFS median: 4.0 months	[29]
				OS median: 6.9 months	

Abbreviations: BSC, best standard of care; FF, folinic acid and fluorouracil; OFF, oxaliplatin and FF; OS, overall survival; OS12, 12 month overall survival; ORR, objective response rate; PFS, progression-free survival PS, performance status; P, pancreatic cancer; MPC, metastatic pancreatic cancer; DOR, duration of response; TTP, time to progression disease; ECOG, Eastern Cooperative Oncology Group; Q2W, every 2 weeks; Q4W, every 4 weeks; OD, once daily; BID, orally twice a day.

#### Charite ONKOlogie/CONKO-003-Part 1 STUDY: OFF vs. BSC

In 2009, a phase 2 study evaluated the oxaliplatin, folinic acid, and 5-FU (OFF) regimen as second-line treatment for patients with metastatic PCa after failure of first-line treatment with gemcitabine [28]. The CONKO-003 part 1, NCT00786058, a multicenter phase 3 trial was designed to compare OFF and best supportive care (BSC) [30]. The OFF regimen consisted of a 6-week cycle of oxaliplatin (85 mg/m^2^) on days 8 and 22 and Folinic Acid (200 mg/m^2^) followed by 5-FU infusion (2000 mg/m^2^) administered on days 1, 8, 15, and 22. The primary endpoint was efficacy. The median OS for the sequence gemcitabine-OFF was 9.09 months [95% CI: 6.97 to 11.21] and 7.90 months [95% CI: 4.95 to 10.84] for gemcitabine-BSC (HR 0.50 [95% CI: 0.27 to 0.95], *p* = 0.031) [30].

#### CONKO-003-Part 2 TRIAL: OFF vs. 5-FU/LV (FF)

A randomized, open-label, phase 3 study, CONKO-003 part 2, was conducted to assess the efficacy of removing oxaliplatin from the OFF treatment schedule in patients who have experienced progression during first-line gemcitabine monotherapy [29]. A total of 160 patients were stratified by the presence of metastases, the duration of first-line gemcitabine treatment, and a KPS of at least 70% and were subsequently randomized to receive OFF and BSC or FF and BSC [29]. OFF significantly extended the duration of OS when compared with FF alone (OFF group 5.9 months; 95% CI, 4.1 to 7.4 vs. 3.3 months; 95% CI, 2.7 to 4.0 FF group; HR 0.66; 95% CI, 0.48 to 0.91; log-rank *p* = 0.010). Rates of adverse events were similar between treatment arms, except for grades 1 and 2 neurotoxicity, which were reported in 29 patients (38.2%) and 6 patients (7.1%) in the OFF and FF groups, respectively (*p* < 0.001) [29].

#### NCT01121848: mFOLFOX6 vs. FU/LV

PANCREOX was a phase 3 multicenter trial designed to evaluate the benefit of FU and oxaliplatin administered as modified FOLFOX6 (mFOLFOX6) compared with infusion 5-FU/LV [29]. A total of 108 patients with confirmed advanced PC who were previously treated with gemcitabine therapy and with an ECOG PS of 0–2 were eligible. Unfortunately, no benefit was observed with the addition of oxaliplatin. No difference was observed in PFS (median, 3.1 months mFOLFOX6 vs. 2.9 months FU/LV; *p* = 0.99), and OS was inferior in patients assigned to mFOLFOX6 (median, 6.1 months vs. 9.9 months; *p* = 0.02). This study confirms that infusion 5FU/LV is a reasonable and well-tolerated second-line option for patients with advanced pancreatic cancer with an ECOG PS of 2 or better and who had been previously treated with first-line gemcitabine-based therapy [29].

#### NAPOLI-1: Nanoliposomal Irinotecan vs. FF vs. Nanoliposomal Irinotecan + FF

Between 2012 and 2013, a global, phase 3, randomized, open-label trial at 76 sites in 14 countries assessed the effect of nanoliposomal irinotecan monotherapy or combined with FF in patients with MPC who had already been treated with gemcitabine and had a KPS score of 70% or more [31]. A total of 117 patients received 80 mg/m^2^ of irinotecan in continuous infusion and 400 mg/m^2^ of folinic acid followed by 2,400 mg/m^2^ of fluorouracil for 46 h every 2 weeks. A total of 151 patients received irinotecan 120 mg/m^2^ on monotherapy every 3 weeks. All patients underwent a genotype test (UGT11); those with homozygous results reduced the dose of nanoliposomal irinotecan by 20 mg/m^2^, and after the first cycle of absence of drug-related toxic effects, it was increased to the standard dose. In the third arm, 149 patients received 200 mg/m^2^ of folinic acid followed by fluorouracil for 24 h each week for the first 4 weeks of each 6 week cycle [31]. The median OS in patients assigned nanoliposomal irinotecan plus FF was 6.1 months (95% CI 4.8–8.9) vs. 4.2 months with FF (HR 0.67, 95% CI 0.49 to 0.92; *p* = 0.012. In conclusion, the study shows that treatment with nanoliposomal irinotecan plus FF improved OS in patients previously treated with gemcitabine and with a good PS [31].

The ASCO guidelines published in 2020 recommend fluorouracil plus nanoliposomal irinotecan, or fluorouracil plus irinotecan when the previous combination is not available, as second-line therapy for patients who have received first-line treatment with a gemcitabine-based regimen, an ECOG PS of 0–1, a relatively favorable comorbidity profile, patient preference, and a good support network and access to chemotherapy port management services and infusion pumps [14].

#### KEYNOTE 158 Study: Pembrolizumab for Advanced Non-Colorectal High Microsatellite Instability/Mismatch Repair–Deficient (MSI-H/dMMR) Cancer

This is a non-randomized, open-label, phase 2 study enrolling previously treated MSI-H/dMMR advanced cancer patients (27 different tumor types) to receive pembrolizumab 200 mg administered intravenously every 3 weeks for 35 cycles or until disease progression or unacceptable toxicity. Patients with previous exposure to immunotherapy were excluded. (MMR)/MSI status was determined by examining either the loss of protein expression by immunohistochemistry of four MMR enzymes (MLH1/MSH2/MSH6/PMS2) or the analysis of tumor microsatellites using polymerase chain reaction (PCR). The primary endpoint was ORR. Of 233 patients, 23 (9.9%) had a confirmed complete response and 57 (24.5%) had a confirmed partial response. A total of 22 patients in this study had pancreatic cancer (9.4%). Just 1 patient had a complete response and 3 patients had a partial response. The PFS was 2.1 months and the OS was 4 months [13]. New randomized studies comparing this option with current treatments are needed to better understand the role of immunotherapy in this population.

#### Sotorasib in KRAS p.G12C–Mutated Advanced Pancreatic Cancer

This is a phase 1–2, single-arm study. A total of 38 patients with KRAS p.G12C-mutated advanced PC who had received at least one previous systemic therapy received treatment with Sotorasib at a dose of 960 mg orally once daily. The primary endpoint for phase 2 was a centrally confirmed objective response. The patients had received a median of 2 lines of therapy previously. A total of 8 patients had a centrally confirmed objective response (21%). The median PFS was 4.0 months, and the median overall survival was 6.9 months. Treatment-related grade 3 adverse events were reported in 6 patients (16%). No treatment-related adverse events were fatal or led to treatment discontinuation [32].

#### Ongoing Research Treatments

Newer agents such as LOXO-195 and TPX-00005 are currently being tested, specifically for those patients who may acquire resistance to first-generation TRK inhibitors [14, 33]. Research is also underway to determine the extent of cancer risk associated with PALB2 (partner and localizer of BRCA2), which occurs in 3%–4% of familial pancreatic cancer cases [14, 34]. Promising preclinical studies are currently underway, based on CAR-T-Cell therapy, which is based on the collection of T cells genetically modified to express an antigen-binding domain capable of recognizing and attacking cancer cells [35, 36]. Another treatment strategy is represented by vaccines, emerging as innovative immunotherapies, also evaluated in combination with chemotherapy. One of the best-investigated vaccine strategies is granulocyte-macrophage colony-stimulating factor (GMCSF)-allogeneic pancreatic tumor cells (GVAX) [35, 37].

#### Integrative Supportive Care

Patients with pancreatic cancer should receive early intervention by a palliative care team, with careful assessment of symptom burden, psychological status, and social support network [14]. Early palliative care interventions can have a positive impact on quality of life and adherence to treatments [38]. In patients with advanced pancreatic cancer, palliative intervention is a priority and aggressive management of pain and associated symptoms should be offered [39, 40].Supportive care is centered on the patient but should also include the family and caregivers [41].

Pain is the most commonly described symptom, reported by 75% of patients [42]. Usually, it is in the epigastrium and radiates to the ribs and mid-back. The pathophysiology includes combining neuropathic (perineural infiltration by cancer cells of the peripancreatic nerves involving the celiac and splanchnic plexus) and visceral (tissue destruction and inflammation, pancreatic duct obstruction) mechanisms [39]. Therefore, multimodal pain management should be considered, preferably including powerful opioid medications, neuromodulators, and in some cases, anti-inflammatory drugs for short cycles. Opioids are the mainstay of pharmacologic options in treating pain in patients with pancreatic cancer [40]. Because of the dynamic nature of cancer-associated pain and the substantial variation in individual responsiveness to opioids, there may be a need for ongoing adjustments with close monitoring of outcomes (analgesia, adverse effects, activity, and affect) to achieve an individualized tolerated and effective analgesic response [40]. Pain can also be managed with local procedures targeting the celiac plexus or splanchnic nerves, with the benefit of pain control with less opioid consumption [30, 39]. Radiotherapy is an option for the management of severe pain or bleeding [43]. Other symptoms related to digestive obstructions (gastric outlet, bile duct, duodenal) cause jaundice, pruritus, and an increased risk for cholangitis; options for palliation include endoscopic metal stents [44] and surgical bypass, which can often be performed laparoscopically reserved in those for whom stent placement is not possible due to technical reasons and in those found to be unresectable at the time of operative exploration [45].

Among further topics, nutrition counseling should be provided to all patients to stimulate appetite and prevent cancer-related anorexia-cachexia syndrome, deconditioning, and cancer-related fatigue [46, 47].

## Discussion

Advanced PC remains a poor prognosis disease with no curative treatment options, with new trials failing to change first-line treatment. However, knowledge of the molecular biology of the tumor has offered a new way of understanding the disease. Even though the cases with molecular targets represent a small portion of patients, molecular targeted therapy seems to be the best available approach to try to give our patients a better quality of life. Today, it is essential to identify these subgroups that can be treated differentially to optimize treatment and reduce toxicity.

Although first-line and second-line chemotherapy regimens have not changed significantly in recent years, it is important to mention that there are new options, such as liposomal irinotecan, that have demonstrated survival advantages; however, its high cost makes it difficult for it to be widely adopted by most countries. In this review, we identify clear examples of molecular targeted therapy that have recently entered clinical practice, which include the use of inhibitors of PARP (IPARP) for PC with mutation in the BRCA genes and immunotherapy for PC with MSI-H/dMMR.

POLO 1 demonstrated a longer PFS (7.4 months vs. 3.8 months) in BRCA-mutated patients treated with platinum-based chemotherapy followed by Olaparib, while the OS difference was not statistically significant. The BRCA-mutant PC population represents approximately 5% of all pancreatic adenocarcinomas [48]. It is important to know the BRCA status early to use platinum as first-line therapy for the mutated population. BRCA-mutant cancers are known to have high sensitivity to platins because these tumors cannot adequately resolve platinum-induced DNA damage [49]. However, limited access to IPARPs in most countries hinders the clinical applicability of this therapy.

On the other hand, tumors with dMMR account for approximately 2%–4% of all cancers. Cells from dMMR tumors may express PD-L1 on their membrane and have many peritumoral infiltrating lymphocytes and high production of mutant protein neoantigens. This phenotype suggests that these tumors are highly sensitive to immune checkpoint blockade [13]. Immunotherapy is approved for any MSI-H/dMMR solid tumor with prior chemotherapy treatment, based on consistent results for this population. The data are limited by the low number of patients with PC in these studies, and it is, therefore, difficult to draw definitive conclusions. Despite this, immunotherapy is certainly a reasonable option for stage IV pancreatic cancer previously treated with standard therapy and MSI-H/dMMR tumors.

The new KRAS inhibitor Sotorasib showed promising results for the pretreated PC KRAS G12C mutated population in a phase 1–2 trial, with a 21% objective response rate, opening the debate on the need for massive next-generation sequencing panels that also include this mutation or others that also predict response to targeted therapies that are still in phase 1 and 2 studies.

## Conclusion

There is a clear clinical need for new treatment strategies that facilitate the improvement of patient outcomes. The molecule-directed approach, although not an alternative for all patients, is the gold standard for cases with validated molecular biomarkers. The search for new biomarkers and new clinical trials is warranted. There are several ongoing studies that could potentially change clinical practice in the coming years.

## Abbreviations

ASCO, American Society of Clinical Oncology; BSC, best supportive care; CT, computed tomography; DOR, duration of response; ECOG, Eastern Cooperative Oncology Group Performance Status; KPS, Karnofsky Performance Status; LS, Lynch syndrome; MPC, metastatic pancreatic cancer; MRI, magnetic resonance imaging; ORR, Objective tumor response rate; OS, overall survival; PC, Pancreatic cancer; PS, Performance Status; PFS, progression-free survival; PCR, polymerase chain reaction; QoL, quality of life; SCNAs, somatic copy number alterations; 5-FU, 5-Fluorouracil; TTP, time to disease progression; WHO, World Health Organization.

## References

[1] Bray F Ferlay J Soerjomataram I Siegel RL Torre LA Jemal A. Global Cancer Statistics 2018: GLOBOCAN Estimates of Incidence and Mortality Worldwide for 36 Cancers in 185 Countries. CA Cancer J Clin (2018) 68(6):394–424. 10.3322/caac.21492 30207593

[2] Stoffel EM Brand RE Goggins M. Pancreatic Cancer: Changing Epidemiology and New Approaches to Risk Assessment, Early Detection, and Prevention. Gastroenterology (2023) 164(5):752–65. 10.1053/j.gastro.2023.02.012 36804602 PMC10243302

[3] Park W Chawla A O’Reilly EM. Pancreatic Cancer: A Review. JAMA - J Am Med Assoc (2021) 326:851–62. 10.1001/jama.2021.13027 PMC9363152 34547082

[4] Klein AP. Pancreatic Cancer Epidemiology: Understanding the Role of Lifestyle and Inherited Risk Factors. Nat Rev Gastroenterol Hepatol (2021) 18:493–502. Nature Research. 10.1038/s41575-021-00457-x 34002083 PMC9265847

[5] de Dosso S Siebenhüner AR Winder T Meisel A Fritsch R Astaras C Treatment Landscape of Metastatic Pancreatic Cancer. Cancer Treat Rev (2021) 96:102180. 10.1016/j.ctrv.2021.102180 33812339

[6] Karakas Y Lacin S Yalcin S. Recent Advances in the Management of Pancreatic Adenocarcinoma. Expert Rev Anticancer Ther (2018) 18:51–62. 10.1080/14737140.2018.1403319 29125367

[7] Ettrich TJ Seufferlein T. Systemic Therapy for Metastatic Pancreatic Cancer. Curr Treat Options Oncol (2021) 22(11):106. 10.1007/s11864-021-00895-4 34665339 PMC8526424

[8] Jones S Zhang X Parsons DW Lin JCH Leary RJ Angenendt P Core Signaling Pathways in Human Pancreatic Cancers Revealed by Global Genomic Analyses. Science (2008) 321(5897):1801–6. 10.1126/science.1164368 18772397 PMC2848990

[9] Luo J. KRAS Mutation in Pancreatic Cancer. Semin Oncol (2021) 48:10–8. 10.1053/j.seminoncol.2021.02.003 33676749 PMC8380752

[10] Salem ME El-Refai SM Sha W Puccini A Grothey A George TJ Landscape of KRAS G12C, Associated Genomic Alterations, and Interrelation With Immuno-Oncology Biomarkers in KRAS -Mutated Cancers. JCO Precision Oncol (2022) 6(6):e2100245. 10.1200/po.21.00245 PMC8966967 35319967

[11] Raphael BJ Hruban RH Aguirre AJ Moffitt RA Yeh JJ Stewart C Integrated Genomic Characterization of Pancreatic Ductal Adenocarcinoma. Cancer Cell (2017) 32(2):185–203.e13. 10.1016/j.ccell.2017.07.007 28810144 PMC5964983

[12] Hendifar AE Larson BK Rojansky R Guan M Gong J Placencio V Pancreatic Cancer “Mismatch” in Lynch Syndrome. BMJ Open Gastroenterol (2019) 6(1):e000274. 10.1136/bmjgast-2019-000274 PMC6577306 31275582

[13] Marabelle A Le DT Ascierto PA Di Giacomo AM de Jesus-Acosta A Delord JP Efficacy of Pembrolizumab in Patients With Noncolorectal High Microsatellite Instability/Mismatch Repair–Deficient Cancer: Results From the Phase II KEYNOTE-158 Study. J Clin Oncol (2020) 38(1):1–10. 10.1200/jco.19.02105 31682550 PMC8184060

[14] Sohal DPS Kennedy EB Cinar P Conroy T Copur MS Crane CH Metastatic Pancreatic Cancer: ASCO Guideline Update. J Clin Oncol (2020) 38:3217–30. 10.1200/jco.20.01364 32755482 PMC12974607

[15] Hohla F Hopfinger G Romeder F Rinnerthaler G Bezan A Stättner S Female Gender May Predict Response to FOLFIRINOX in Patients With Unresectable Pancreatic Cancer: A Single Institution Retrospective Review. Int J Oncol (2014) 44(1):319–26. 10.3892/ijo.2013.2176 24247204

[16] Lambert A Jarlier M Gourgou Bourgade S Conroy T. Response to FOLFIRINOX by Gender in Patients With Metastatic Pancreatic Cancer: Results From the PRODIGE 4/ACCORD 11 Randomized Trial. PLoS One (2017) 12(9):e0183288. 10.1371/journal.pone.0183288 28931010 PMC5606928

[17] Ueno H Ioka T Ikeda M Ohkawa S Yanagimoto H Boku N Randomized Phase Iii Study of Gemcitabine Plus S-1, S-1 Alone, or Gemcitabine Alone in Patients With Locally Advanced and Metastatic Pancreatic Cancer in Japan and Taiwan: Gest Study. J Clin Oncol (2013) 31:1640–8. 10.1200/jco.2012.43.3680 23547081

[18] Okusaka T Miyakawa H Fujii H Nakamori S Satoh T Hamamoto Y Updated Results From GEST Study: A Randomized, Three-Arm Phase III Study for Advanced Pancreatic Cancer. J Cancer Res Clin Oncol (2017) 143(6):1053–9. 10.1007/s00432-017-2349-y 28210843 PMC5427167

[19] Von Hoff DD Ervin T Arena FP Chiorean EG Infante J Moore M Increased Survival in Pancreatic Cancer With Nab-Paclitaxel Plus Gemcitabine. New Engl J Med (2013) 369(18):1691–703. 10.1056/nejmoa1304369 24131140 PMC4631139

[20] Cunningham D Chau I Stocken DD Valle JW Smith D Steward W Phase III Randomized Comparison of Gemcitabine versus Gemcitabine Plus Capecitabine in Patients With Advanced Pancreatic Cancer. J Clin Oncol (2009) 27(33):5513–8. 10.1200/jco.2009.24.2446 19858379

[21] O’Hara MH O’Reilly EM Varadhachary G Wolff RA Wainberg ZA Ko AH CD40 Agonistic Monoclonal Antibody APX005M (Sotigalimab) and Chemotherapy, With or Without Nivolumab, for the Treatment of Metastatic Pancreatic Adenocarcinoma: An Open-Label, Multicentre, Phase 1b Study. Lancet Oncol (2021) 22(1):118–31. 10.1016/S1470-2045(20)30532-5 33387490

[22] Sultana A Smith CT Cunningham D Starling N Neoptolemos JP Ghaneh P. Meta-Analyses of Chemotherapy for Locally Advanced and Metastatic Pancreatic Cancer. J Clin Oncol (2007) 25:2607–15. 10.1200/jco.2006.09.2551 17577041

[23] Burris HA Moore MJ Andersen J Green MR Rothenberg ML Modiano MR Improvements in Survival and Clinical Benefit With Gemcitabine as First-Line Therapy for Patients With Advanced Pancreas Cancer: A Randomized Trial. J Clin Oncol (1997) 15(6):2403–13. 10.1200/jco.1997.15.6.2403 9196156

[24] Moore MJ Goldstein D Hamm J Figer A Hecht JR Gallinger S Erlotinib Plus Gemcitabine Compared With Gemcitabine Alone in Patients With Advanced Pancreatic Cancer: A Phase III Trial of the National Cancer Institute of Canada Clinical Trials Group. J Clin Oncol (2007) 25(15):1960–6. 10.1200/jco.2006.07.9525 17452677

[25] Conroy T Desseigne F Ychou M Bouché O Guimbaud R Bécouarn Y FOLFIRINOX Versus Gemcitabine for Metastatic Pancreatic Cancer. New Engl J Med (2011) 364(19):1817–25. 10.1056/nejmoa1011923 21561347

[26] Padrón LJ Maurer DM O’Hara MH O’Reilly EM Wolff RA Wainberg ZA Sotigalimab And/or Nivolumab With Chemotherapy in First-Line Metastatic Pancreatic Cancer: Clinical and Immunologic Analyses From the Randomized Phase 2 PRINCE Trial. Nat Med (2022) 28(6):1167–77. 10.1038/s41591-022-01829-9 35662283 PMC9205784

[27] Wainberg ZA Melisi D Macarulla T Pazo Cid R Chandana SR De La Fouchardière C NALIRIFOX Versus Nab-Paclitaxel and Gemcitabine in Treatment-Naive Patients With Metastatic Pancreatic Ductal Adenocarcinoma (NAPOLI 3): A Randomised, Open-Label, Phase 3 Trial. The Lancet (2023) 402:1272–81. 10.1016/s0140-6736(23)01366-1 PMC11664154 37708904

[28] Pelzer U Stieler J Roll L Hilbig A Dörken B Riess H Second-Line Therapy in Refractory Pancreatic Cancer. Results of a Phase Ii Study. Oncol Res Treat (2009) 32(3):99–102. 10.1159/000197769 19295247

[29] Gill S Ko YJ Cripps C Beaudoin A Dhesy-Thind S Zulfiqar M PANCREOX: A Randomized Phase III Study of Fluorouracil/leucovorin With or Without Oxaliplatin for Second-Line Advanced Pancreatic Cancer in Patients Who Have Received Gemcitabine-Based Chemotherapy. J Clin Oncol (2016) 34(32):3914–20. 10.1200/jco.2016.68.5776 27621395

[30] Pelzer U Schwaner I Stieler J Adler M Seraphin J Dörken B Best Supportive Care (BSC) Versus Oxaliplatin, Folinic Acid and 5-Fluorouracil (OFF) Plus BSC in Patients for Second-Line Advanced Pancreatic Cancer: A Phase III-Study From the German CONKO-Study Group. Eur J Cancer (2011) 47(11):1676–81. 10.1016/j.ejca.2011.04.011 21565490

[31] Wang-Gillam A Li CP Bodoky G Dean A Shan YS Jameson G Nanoliposomal Irinotecan With Fluorouracil and Folinic Acid in Metastatic Pancreatic Cancer After Previous Gemcitabine-Based Therapy (NAPOLI-1): A Global, Randomised, Open-Label, Phase 3 Trial. The Lancet (2016) 387(10018):545–57. 10.1016/s0140-6736(15)00986-1 26615328

[32] Strickler JH Satake H George TJ Yaeger R Hollebecque A Garrido-Laguna I Sotorasib in KRAS p.G12C–Mutated Advanced Pancreatic Cancer. New Engl J Med (2023) 388(1):33–43. 10.1056/nejmoa2208470 36546651 PMC10506456

[33] Cocco E Scaltriti M Drilon A. NTRK Fusion-Positive Cancers and TRK Inhibitor Therapy. Nat Rev Clin Oncol (2018) 15:731–47. Nature Publishing Group. 10.1038/s41571-018-0113-0 30333516 PMC6419506

[34] Hofstatter EW Domchek SM Miron A Garber J Wang M Componeschi K PALB2 Mutations in Familial Breast and Pancreatic Cancer. Fam Cancer (2011) 10(2):225–31. 10.1007/s10689-011-9426-1 21365267 PMC3836668

[35] Balsano R Zanuso V Pirozzi A Rimassa L Bozzarelli S. Pancreatic Ductal Adenocarcinoma and Immune Checkpoint Inhibitors: The Gray Curtain of Immunotherapy and Spikes of Lights. Curr Oncol (2023) 30:3871–85. MDPI. 10.3390/curroncol30040293 37185406 PMC10136659

[36] Leidner R Sanjuan Silva N Huang H Sprott D Zheng C Shih YP Neoantigen T-Cell Receptor Gene Therapy in Pancreatic Cancer. New Engl J Med (2022) 386(22):2112–9. 10.1056/nejmoa2119662 35648703 PMC9531755

[37] Le DT Wang-Gillam A Picozzi V Greten TF Crocenzi T Springett G Safety and Survival With GVAX Pancreas Prime and Listeria Monocytogenes-Expressing Mesothelin (CRS-207) Boost Vaccines for Metastatic Pancreatic Cancer. J Clin Oncol (2015) 33(12):1325–33. 10.1200/jco.2014.57.4244 25584002 PMC4397277

[38] Chung V Sun V Ruel N Smith TJ Ferrell BR. Improving Palliative Care and Quality of Life in Pancreatic Cancer Patients. J Palliat Med (2022) 25(5):720–7. 10.1089/jpm.2021.0187 34704841 PMC9080991

[39] Drewes AM Campbell CM Ceyhan GO Delhaye M Garg PK van Goor H Pain in Pancreatic Ductal Adenocarcinoma: A Multidisciplinary, International Guideline for Optimized Management. Pancreatology (2018) 18:446–57. 10.1016/j.pan.2018.04.008 29706482

[40] Coveler AL Mizrahi J Eastman B Apisarnthanarax SJ Dalal S McNearney T Pancreas Cancer-Associated Pain Management. The Oncologist (2021) 26:e971–82. John Wiley and Sons Inc. 10.1002/onco.13796 33885205 PMC8176967

[41] Laquente B Calsina-Berna A Carmona-Bayonas A Jiménez-Fonseca P Peiró I Carrato A. Supportive Care in Pancreatic Ductal Adenocarcinoma. Clin Translational Oncol (2017) 19:1293–302. 10.1007/s12094-017-1682-6 28612201

[42] Varghese AM Lowery MA Yu KH O’Reilly EM. Current Management and Future Directions in Metastatic Pancreatic Adenocarcinoma. Cancer (2016) 122:3765–75. 10.1002/cncr.30342 27649047 PMC5565512

[43] Habermehl D Brecht IC Debus J Combs SE. Palliative Radiation Therapy in Patients With Metastasized Pancreatic Cancer - Description of a Rare Patient Group. Eur J Med Res (2014) 19(1):24. 10.1186/2047-783x-19-24 24887532 PMC4046029

[44] Moss A Morris E Mac Mathuna P. Palliative Biliary Stents for Obstructing Pancreatic Carcinoma. Cochrane database Syst Rev (2006) 1:CD004200. John Wiley & Sons, Ltd. 10.1002/14651858.CD004200.pub2 16437477

[45] Ma KW Chan ACY She WH Chok KSH Dai WC Tsang S Efficacy of Endoscopic Self-Expandable Metal Stent Placement Versus Surgical Bypass for Inoperable Pancreatic Cancer-Related Malignant Biliary Obstruction: A Propensity Score-Matched Analysis. Surg Endosc (2018) 32(2):971–6. 10.1007/s00464-017-5774-8 28779260

[46] Tempero MA Malafa MP Al-Hawary M Behrman SW Benson AB Cardin DB Pancreatic Adenocarcinoma, Version 2.2021, NCCN Clinical Practice Guidelines in Oncology. J Natl Compr Cancer Netw (2021) 19(4):439–57. 10.6004/jnccn.2021.0017 33845462

[47] Muscaritoli M Arends J Bachmann P Baracos V Barthelemy N Bertz H ESPEN Practical Guideline: Clinical Nutrition in Cancer. Clin Nutr (2021) 40:2898–913. 10.1016/j.clnu.2021.02.005 33946039

[48] Holter S Borgida A Dodd A Grant R Semotiuk K Hedley D Germline BRCA Mutations in a Large Clinic-Based Cohort of Patients With Pancreatic Adenocarcinoma. J Clin Oncol (2015) 33:3124–9. 10.1200/jco.2014.59.7401 25940717

[49] Mylavarapu S Das A Roy M. Role of BRCA Mutations in the Modulation of Response to Platinum Therapy. Front Oncol (2018) 8:16. 10.3389/fonc.2018.00016 29459887 PMC5807680

